# Functional Study of Lipoxygenase-Mediated Resistance against *Fusarium verticillioides* and *Aspergillus flavus* Infection in Maize

**DOI:** 10.3390/ijms231810894

**Published:** 2022-09-17

**Authors:** Mikias Damtew Guche, Stefania Pilati, Francesco Trenti, Lorenza Dalla Costa, Paola Giorni, Graziano Guella, Adriano Marocco, Alessandra Lanubile

**Affiliations:** 1 Department of Sustainable Crop Production, Università Cattolica del Sacro Cuore, Via Emilia Parmense 84, 29122 Piacenza, Italy; 2 C3A—Centro Agricoltura Alimenti Ambiente, Via Edmund Mach 1, 38098 San Michele all’Adige, Italy; 3 Research and Innovation Centre, Fondazione Edmund Mach, Via E. Mach 1, 38098 San Michele all’Adige, Italy; 4 Department of Physics, University of Trento, Via Sommarive 14, 38123 Povo, Italy

**Keywords:** lipoxygenase, pan-genomes, pan-transcriptomes, fungal diseases, fumonisins, aflatoxins, 10-OPEA, *Zea mays*

## Abstract

Mycotoxin contamination of maize kernels by fungal pathogens like *Fusarium verticillioides* and *Aspergillus flavus* is a chronic global challenge impacting food and feed security, health, and trade. Maize *lipoxygenase* genes (*ZmLOXs*) synthetize oxylipins that play defense roles and govern host-fungal interactions. The current study investigated the involvement of *ZmLOXs* in maize resistance against these two fungi. A considerable intraspecific genetic and transcript variability of the ZmLOX family was highlighted by in silico analysis comparing publicly available maize pan-genomes and pan-transcriptomes, respectively. Then, phenotyping and expression analysis of *ZmLOX* genes along with key genes involved in oxylipin biosynthesis were carried out in a maize mutant carrying a Mu transposon insertion in the *ZmLOX4* gene (named UFMu*lox4*) together with Tzi18, Mo17, and W22 inbred lines at 3- and 7-days post-inoculation with *F. verticillioides* and *A. flavus*. Tzi18 showed the highest resistance to the pathogens coupled with the lowest mycotoxin accumulation, while UFMu*lox4* was highly susceptible to both pathogens with the most elevated mycotoxin content. *F. verticillioides* inoculation determined a stronger induction of *ZmLOXs* and maize *allene oxide synthase* genes as compared to *A. flavus*. Additionally, oxylipin analysis revealed prevalent linoleic (18:2) peroxidation by 9-LOXs, the accumulation of 10-oxo-11-phytoenoic acid (10-OPEA), and triglyceride peroxidation only in *F. verticillioides* inoculated kernels of resistant genotypes.

## 1. Introduction

Mycotoxin contamination of maize kernels by the fungal pathogens *Fusarium verticillioides* and *Aspergillus flavus* is a chronic global challenge throughout the maize value chain affecting food and feed security, health, and trade [1,2]. Moreover, the production of fumonisins and aflatoxins by *F. verticillioides* and *A. flavus*, respectively, is expected to be exacerbated by the current climate change scenario worldwide [3,4]. In addition, a large portion of the developing world is lagging with the implementation of a well-designed mycotoxin regulation system [5].

Several disease management strategies were exploited by maize growers to control these pathogens, including the adoption of good crop management practices [6] and biological control using non-toxigenic strains [7]. 

However, these strategies alone can be inadequate and, thus, host-mediated resistance can be an effective and sustainable means for the integrated management of fungal diseases. In this regard, the understanding of the molecular intracellular signaling cascade of maize-mycotoxigenic fungi interactions is crucial to finding better sources of disease resistance in this crop. *F. verticillioides* is a hemibiotrophic pathogen, with an initial biotrophic phase followed by a necrotrophic behavior [8], whereas *A. flavus* is mainly a saprophytic fungus acting as a necrotroph [9]. Although the two fungi are the causal agents of Fusarium ear rot (FER) and Aspergillus ear rot (AER), respectively, they also attack other maize organs at different growth stages, such as seedling, root, and stalk [8,9]. 

Generally, commercial maize cultivars are susceptible to these pathogens, but several attempts to achieve maize lines with increased resistance to *F. verticillioides* [10,11] and *A. flavus* [11,12] have been reported in the literature. However, the resistance trait’s complex genetic architecture, which is frequently associated with undesirable agronomic characteristics, has hampered its widespread adoption [13,14,15,16,17,18,19]. 

During local and systemic plant responses to fungal pathogen infection, lipid peroxidation takes place and initiates the synthesis of different compounds known as oxylipins [20,21]. The first step of oxylipin synthesis is catalyzed by lipoxygenases (LOXs), a family of nonheme iron-containing ubiquitous enzymes involved in the peroxidation of free polyunsaturated fatty acids (PUFA), predominantly linoleic (18:2) or linolenic acid (18:3), but also esterified lipids such as galactolipids and triacylglycerols [22,23]. Downstream of the LOX catalyzed peroxidation step, the oxylipin pathway becomes highly branched: hydroperoxylyase (HPL) activity brings to the production of green leaf volatiles (GLVs), allene oxide synthases (AOS) and cyclase (AOC) acting on linolenic acid catalyze the synthesis of oxo-phytodienoic acid (OPDA) and jasmonic acid (JA), and divinyl ether synthase (DES) drives the synthesis of divinyl ether PUFAs, such as etherolenic acid [24]. Other branches are catalyzed by LOX itself, namely peroxygenases or reductases [22,25]. Intracellular compartmentation as well as gene expression regulation orchestrate the balance of the different biosynthetic pathways.

The maize genome encodes thirteen LOX isoforms (ZmLOXs) grouped into 9- and 13-LOX subfamilies [21,25]. Previous studies established localization and function for some maize LOXs. Concerning 13-LOX, ZmLOX8 was detected in the chloroplasts and found responsible for JA synthesis [25], while ZmLOX10 was localized in the cytosol and involved in GLVs [26]. 9-LOXs are a more heterogeneous group [27], as they are found in the cytoplasm, such as ZmLOX2, or aggregated in vesicle-like structures, such as ZmLOX12, or in the vacuole as tonoplast associated, as in the case of ZmLOX4. ZmLOX6, which showed an activity similar to hydroperoxide lyase, was visualized in the chloroplasts [28]. The attribution of specific classes of oxylipins to specific single 9-LOX has not been possible so far due to a high degree of redundancy. One interesting metabolite with both fungitoxic activity and signaling effects is 10-oxo-11-phytoenoic acid (10-OPEA) [29,30]. It requires the activity of a 9-LOX, non-chloroplastic 9-AOS, and allene oxide cyclase (AOC). The latter has not been yet identified but the existence of such AOC activity has been suggested [31], while a mixed 9/13 AOS has been identified [31] and it was upregulated in southern leaf blight caused by *Cochliobolus heterostrophus* in maize, together with 10-OPEA accumulation [29]. A recent transcriptomic and metabolomic study highlighted the complexity of this pathway, showing that resistance could be related to a plethora of 9-LOX derived compounds, among which, but not restricted to, 10-OPEA [32].

Several authors attempted to better elucidate the LOX-mediated plant defense response against *F. verticillioides* and *A. flavus* infection by employing several inbred lines and *lox* knock-out mutants as well [33,34,35,36,37,38,39]. In the current study, the role of ZmLOXs was further investigated using in silico and in planta approaches. The genetic intraspecific variability, protein domain structures, and basal transcript abundance were explored by comparing publicly available maize pan-genomes and pan-transcriptomes. Moreover, through the exploitation of a maize mutant carrying a Mu transposon insertion in the exon 9 of the *ZmLOX4* gene (hereafter named UFMu*lox4*), the involvement of ZmLOXs against *F. verticillioides* and *A. flavus* infection was explored. Previous studies highlighted that the deletion in the first exon of this gene impaired resistance to *F. verticillioides* and altered *ZmLOX* gene expression as well as the LOX enzymatic activity in wounded kernels in vitro [39] and seedlings in vivo [38]. This study provides the first insight into *ZmLOX* modulation in the UFMu*lox4* mutant together with Tzi18, Mo17, and W22 inbred lines tested in field conditions at 3- and 7-days post-inoculation (dpi) with *F. verticillioides* and *A. flavus*. The integration of expression data with phenotyping and oxylipin profiling analysis supported the requirement of ZmLOX4 for controlling resistance mechanisms against FER and AER. 

## 2. Results

### 2.1. Overview of the LOX Family in Maize Pan-Genome and Pan-Transcriptome

Several maize line genomes are now publicly available at the Maize Genetics and Genomics Database (MaizeGDB) [40] and can be used to investigate intraspecific diversity at different levels, from gene structure, sequence, and methylation to their expression level. In this study, it was estimated the intraspecific divergence within the ZmLOX gene family at the entire protein and functional domain level and at the transcriptional level. After the identification of 13 ZmLOX gene models in the B73_v5 reference genome assembly [41,42], including six 9-LOXs, six 13-LOXs, and the ZmLOX6 lyase, they were searched in the genomes of 25 Nested Association Mapping (NAM) founder lines, representing valuable and well-characterized maize germplasm for several traits ranging from agronomic characteristics to ionomics profiles [43], and additional key inbreds including Mo17, W22, PH207, EP1, and F7, publicly available at the maizeGDB database. For each ZmLOX allozyme, protein sequences were aligned, and the domain structure was analyzed and described in Figures S1 and S2. Presence Absence Variations (PAVs) were found among the 9-LOX gene models, with ZmLOX2 missing in PH207, ZmLOX3 missing in TX303, ZmLOX4 missing in CML277, CML333, and PH207, and ZmLOX12 missing in CML228 and CML247. In addition, a duplication event was observed for ZmLOX3 in PH207.

Despite a general conservation of the protein organization in the two PLAT/LH2 and lipoxygenase domains, variants were identified: some lacked the PLAT/LH2 domain, while others were prematurely truncated at the C-terminal end. The latter isoforms may show defective functionality when the terminal isoleucine is missing, as it is required to coordinate the catalytic iron in the active site, whereas the former isoforms may be impacted in protein-protein or protein-membrane interaction. Interestingly, ZmLOX13 seemed to lack the PLAT/LH2 domain in all the genotypes, as well as the lyase ZmLOX6. ZmLOX6 had a C-terminal valine residue instead of the above-mentioned isoleucine [28], which was conserved in all the inbred lines. 

Due to the two PAVs (ZmLOX2 and ZmLOX4), one gene duplication (ZmLOX3), and many predicted non-functional alleles (ZmLOX1, 5, 8, and 9), the PH207 genotype was the most divergent one. However, experimental validation of the predicted gene models in all the genotypes would be required before drawing any conclusion.

Focusing on the genotypes used in this study, it was observed that W22 lacked a functional ZmLOX12, and Mo17 had a quite different ZmLOX6 structure as some additional domains (FGAR-AT_N, FGAR-AT_linker, PurM-like, and GAT_1 superfamily domains) were gained at the C-terminal, which could alter its activity (Figures S1 and S2).

**Figure 1:**
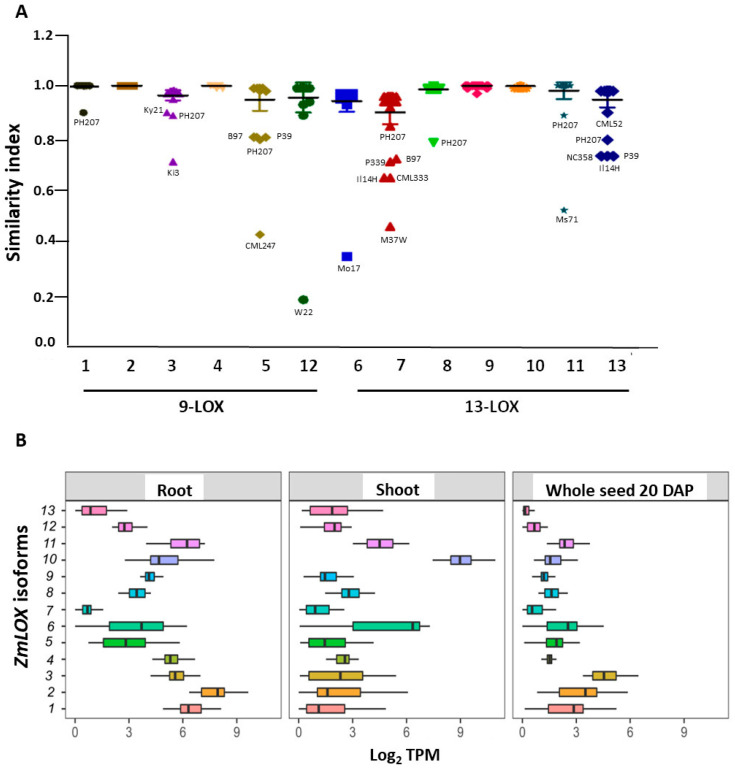
In silico analysis of maize intraspecific ZmLOX protein sequence diversity and tissue-specific gene expression. (**A**) Global Blosum62 similarity index of ZmLOX protein sequences derived from B73_v5, the 25 NAM founders and Mo17, W22, PH207, EP1, and F7 inbred line genomes available at maizeGDB. The horizontal bar indicates the mean value of the similarity index for each ZmLOX. (**B**) Basal *ZmLOX* transcript abundance in the root, shoot, and whole seed at 20 days after pollination (DAP). Data were extracted from transcriptomic datasets of B73_v5 and 25 NAM lines available at maizeGDB and expressed as log_2_ transcripts per million (TPM). In the graphs, vertical lines within boxes indicate the median, and boxes indicate the upper (75%) and lower (25%) quartiles. Whiskers indicate the range of the minimum and maximum values.

The structural and sequence differences of ZmLOXs in the different genetic backgrounds were summarized in Figure 1A, by using a Blosum62-derived global similarity matrix based on ZmLOX alignments. It can be appreciated that some isoforms were highly conserved among all the genotypes, such as the 9-LOXs ZmLOX2 and 4 and the 13-LOX ZmLOX9 and 10, but also ZmLOX1 and 8 were highly conserved, except in the PH207 line, due to C- and N-terminal deletions (Figures S1 and S2), respectively. 

Among the 9-LOXs, ZmLOX3, 5, and 12 showed a higher degree of divergence among the considered lines, as did the 13-LOXs and ZmLOX7, 11, and 13. In general, structural differences had a larger effect on the reduction of the similarity index. 

Finally, the gene expression level of the different allozymes was analyzed in B73 and NAM lines, for which transcriptomic data were available, to gain insight into expression variability. The basal transcript abundance in the root, shoot, and whole seed tissue at 20 days after pollination is reported in Figure 1B. In root samples, the transcripts of 9-LOX isoforms *ZmLOX1*, *2*, *3*, and *4* were abundant, as well as those of *ZmLOX10* and *11*; conversely, in the shoot, *ZmLOX6* and *10* were the most expressed *ZmLOXs*, as expected in aerial plant parts for isoforms involved in volatile production. The seed did not show a high level of *ZmLOX* expression, especially for the 13-LOX group, while among the 9-LOX isoforms, *ZmLOX1*, *2,* and *3* were the most expressed. 

### 2.2. Evaluation of Fungal Growth and Mycotoxin Content

**Figure 2:**
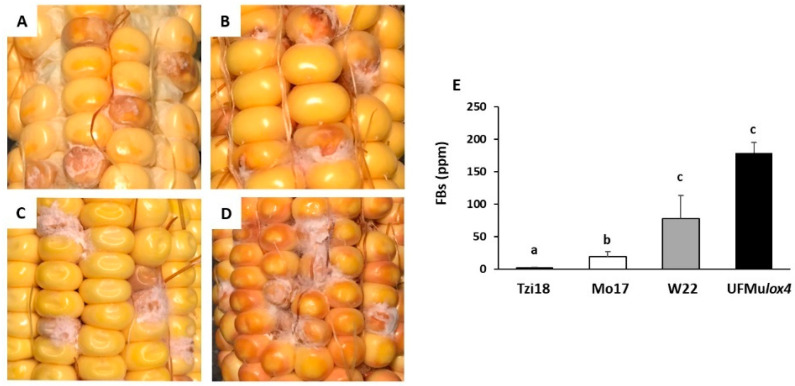
Phenotypic evaluation of Fusarium ear rot disease among the lines (**A**) Tzi18, (**B**) Mo17, (**C**) W22, (**D**) UFMu*lox4* mutant, and (**E**) content of total fumonisins (FBs, B_1_ + B_2_ + B_3_) in artificially inoculated kernels at 7 dpi with *F. verticillioides*. Vertical bars indicate the standard error. The different letters over the histograms indicate significant differences in the fumonisin content among the tested lines as resulting from the Tukey multiple comparison test (*p* ≤ 0.05).

**Figure 3:**
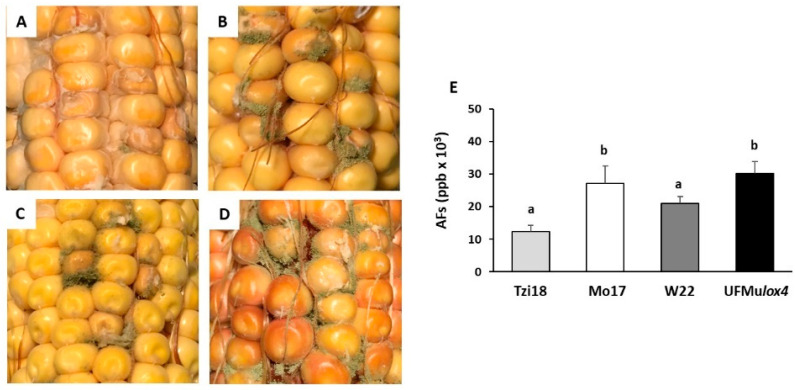
Phenotypic evaluation of Aspergillus ear rot disease among the lines (**A**) Tzi18, (**B**) Mo17, (**C**) W22, (**D**) UFMu*lox4* mutant, and (**E**) content of total aflatoxins (AFs, B_1_ + B_2_ + G_1_ + G_2_) in artificially inoculated kernels at 7 dpi with *A. flavus*. Vertical bars indicate the standard error. The different letters over the histograms indicate significant differences in the aflatoxin content among the tested lines, as resulting from the Tukey multiple comparison test (*p* ≤ 0.05).

To detect whether ZmLOX4 is implicated in the resistance of maize mycotoxigenic fungi, ear rot severity was tested in developing kernels of the UFMulox4 mutant and its wild-type counterpart W22, along with Tzi18 and Mo17 inbred lines at 7 dpi with *F. verticillioides* and *A. flavus*. In addition, in the same samples, mycotoxin content was assessed (Figure 2 and Figure 3, Table S1).

As regards FER, severity symptoms were more circumscribed and limited to the inoculation point in the resistant line Tzi18 (% of FER = 3.5 ± 0.5), where brown discoloration in the pericarp and aleurone layers were observed together with superficial or almost absent fungal growth structures (Figure 2A, Table S1). Intermediate levels of resistance were found for Mo17 (% of FER = 25.5 ± 2.3) (Figure 2B, Table S1), whereas in contrast, inoculated kernels of inbred W22 and UFMu*lox4* mutant were highly affected by the pathogen with extensive fungal structures and conspicuous rotting that also progressed to adjacent kernels, especially in the mutant (% of FER greater than 50.0) (Figure 2C,D, Table S1).

Total fumonisin (B_1_ + B_2_ + B_3_) content was measured in kernels at 7 dpi and results were in line with FER phenotyping (Figure 2E). Fumonisin values ranged from 2.37 ± 0.2 to 179 ± 35.6 ppm for Tzi18 and UFMu*lox4*, respectively. The resistant inbred Tzi18 accumulated significantly (*p* ≤ 0.05) lower fumonisins, followed by Mo17 (19.7 ± 7.4). Contrastingly, the susceptible inbred line W22 and UFMu*lox4* mutant recorded a significantly (*p* ≤ 0.05) higher content of fumonisins. 

Regarding AER, fewer striking differences resulted among inbred lines at 7 dpi, but also, in this case, Tzi18 proved to be the most resistant line, with a reduced growth of *A. flavus* (% of AER = 5.7 ± 1.4), whereas the mutant UFMu*lox4* was highly susceptible (% of AER = 71.4 ± 5.7) (Figure 3, Table S1). Mo17 and W22 showed comparable levels of susceptibility. 

Aflatoxin (B1 + B2 + G1 + G2) content varied from 12.4 × 10^3^ to 30.2 × 10^3^ ppb. The resistant inbred Tzi18 accumulated a relatively lower concentration of aflatoxins that was comparable to W22 (12.4 × 10^3^ and 21 × 10^3^ ppb, respectively). Conversely, significantly (*p* ≤ 0.05) greater production was recorded for the inbred Mo17 (30.2 × 10^3^ ppb) and UFMu*lox4* mutant (27.2 × 10^3^ ppb).

### 2.3. Expression Analysis of Maize Genes Involved in Oxylipin and Jasmonate Synthesis

**Figure 4:**
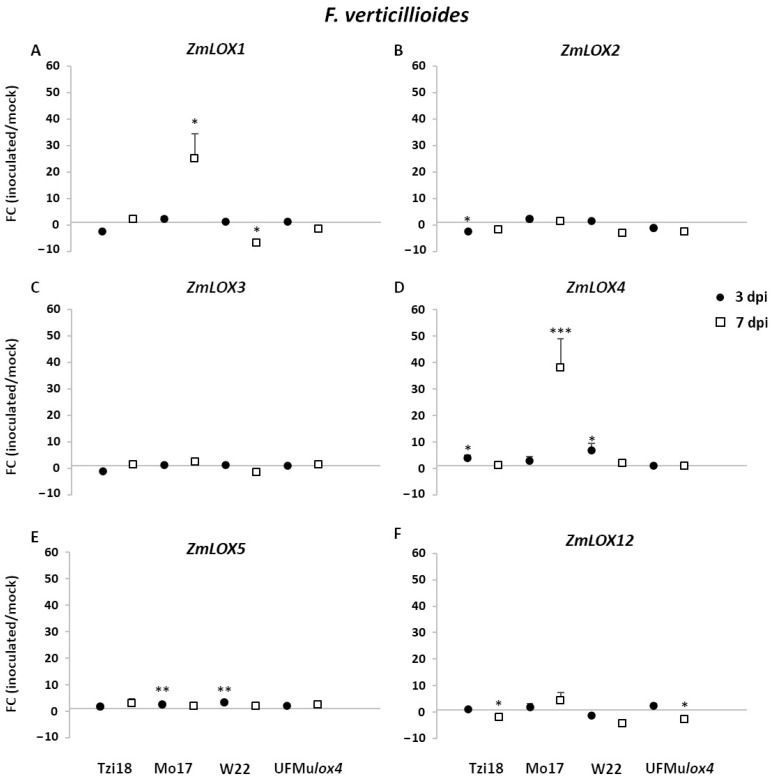
RT-qPCR analysis of maize *9-LOX* genes upon *F. verticillioides* inoculation. Fold change (FC) of expression between inoculated and mock kernels of Tzi18, Mo17, W22, and UFMu*lox4* lines at 3 (circle) and 7 (square) dpi with *F.* *verticillioides*. (**A**) *ZmLOX1*, (**B**) *ZmLOX2*, (**C**) *ZmLOX3*, (**D**) *ZmLOX4*, (**E**) *ZmLOX5,* and (**F**) *ZmLOX12*. Vertical bars indicate the standard error. Asterisk denotes significant upregulation/downregulation with 2-fold or higher FC using Welch’s *t*-test (* *p* ≤ 0.05; ** *p* ≤ 0.01; *** *p* ≤ 0.001).

**Figure 5:**
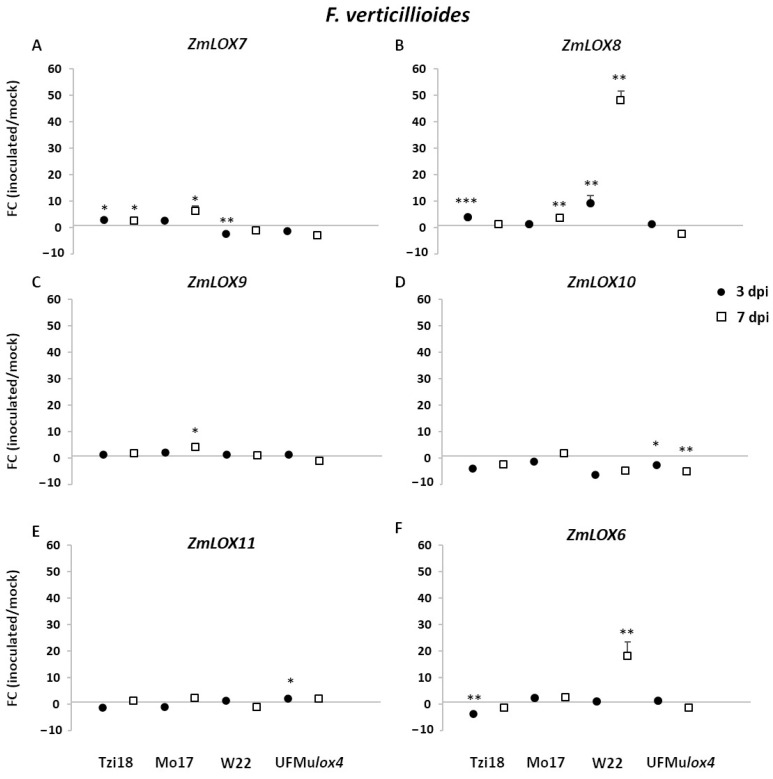
RT-qPCR analysis of maize *13-LOX* genes upon *F. verticillioides* inoculation. Fold change (FC) of expression between inoculated and mock kernels of Tzi18, Mo17, W22, and UFMu*lox4* lines at 3 (circle) and 7 (square) dpi with *F.* *verticillioides*. (**A**) *ZmLOX7*, (**B**) *ZmLOX8*, (**C**) *ZmLOX9*, (**D**) *ZmLOX10*, (**E**) *ZmLOX11,* and (**F**) *ZmLOX6*. Vertical bars indicate the standard error. Asterisk denotes significant upregulation/downregulation with 2-fold or higher FC using Welch’s *t*-test (* *p* ≤ 0.05; ** *p* ≤ 0.01; *** *p* ≤ 0.001).

**Figure 6:**
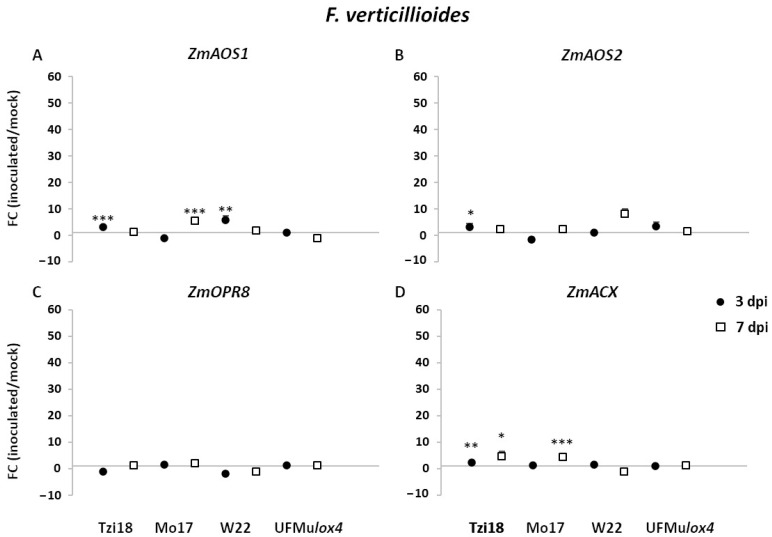
RT-qPCR analysis of maize oxylipin- and JA-related genes upon *F. verticillioides* inoculation. Fold change (FC) of expression between inoculated and mock kernels of Tzi18, Mo17, W22, and UFMu*lox4* lines at 3 (circle) and 7 (square) dpi with *F.* *verticillioides*. (**A**) *ZmAOS1*, (**B**) *ZmAOS2*, (**C**) *ZmOPR8,* and (**D**) *ZmACX*. Vertical bars indicate the standard error. Asterisk denotes significant upregulation/downregulation with 2-fold or higher FC using Welch’s *t*-test (* *p* ≤ 0.05; ** *p* ≤ 0.01; *** *p* ≤ 0.001).

**Figure 7:**
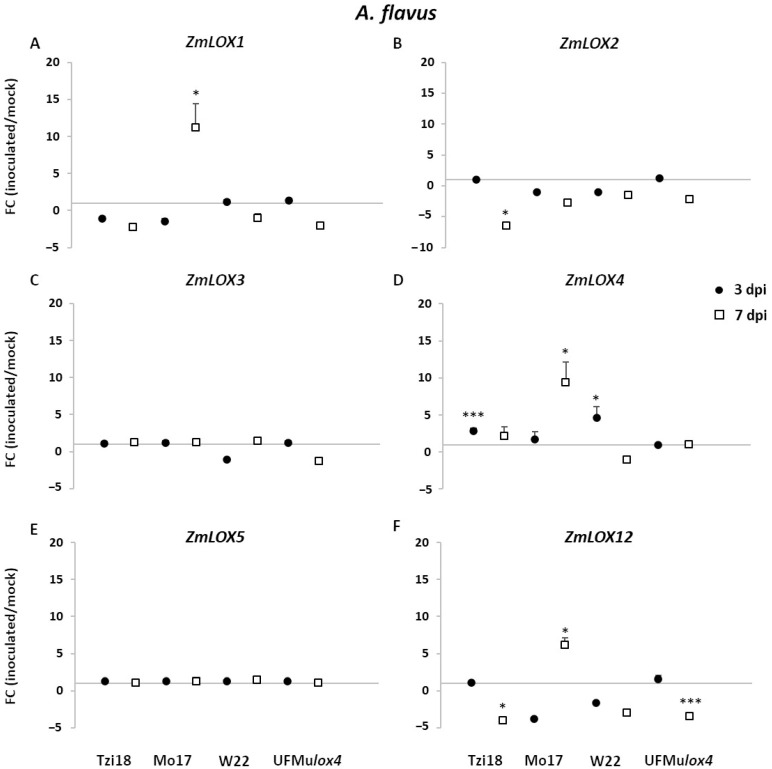
RT-qPCR analysis of maize *9-LOX* genes upon *A. flavus* inoculation. Fold change (FC) of expression between inoculated and mock kernels of Tzi18, Mo17, W22, and UFMu*lox4* lines at 3 (circle) and 7 (square) dpi with *A. flavus*. (**A**) *ZmLOX1*, (**B**) *ZmLOX2*, (**C**) *ZmLOX3*, (**D**) *ZmLOX4*, (**E**) *ZmLOX5,* and (**F**) *ZmLOX12*. Vertical bars indicate the standard error. Asterisk denotes significant upregulation/downregulation with 2-fold or higher FC using Welch’s *t*-test (* *p* ≤ 0.05; *** *p* ≤ 0.001).

**Figure 8:**
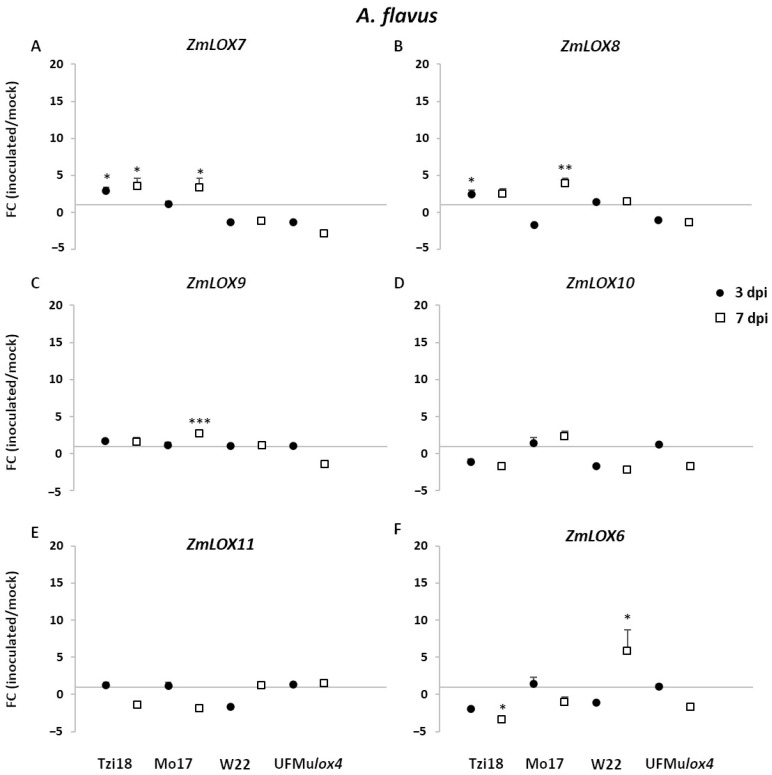
RT-qPCR analysis of maize *13-LOX* genes upon *A. flavus* inoculation. Fold change (FC) of expression between inoculated and mock kernels of Tzi18, Mo17, W22, and UFMu*lox4* lines at 3 (circle) and 7 (square) dpi with *A. flavus*. (**A**) *ZmLOX7*, (**B**) *ZmLOX8*, (**C**) *ZmLOX9*, (**D**) *ZmLOX10*, (**E**) *ZmLOX11*, and (**F**) *ZmLOX6*. Vertical bars indicate the standard error. Asterisk denotes significant upregulation/downregulation with 2-fold or higher FC using Welch’s *t*-test (* *p* ≤ 0.05; ** *p* ≤ 0.01; *** *p* ≤ 0.001).

**Figure 9:**
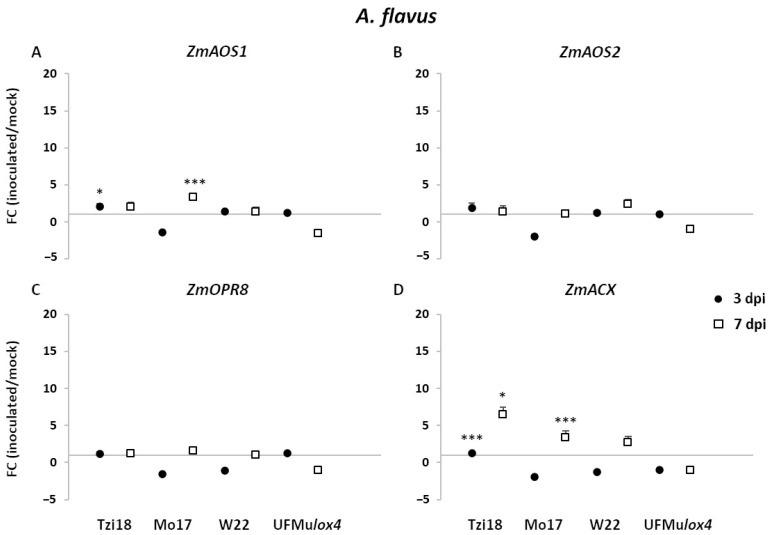
RT-qPCR analysis of maize oxylipin- and JA-related genes upon *A. flavus* inoculation. Fold change (FC) of expression between inoculated and mock kernels of Tzi18, Mo17, W22, and UFMu*lox4* lines at 3 (circle) and 7 (square) dpi with *A. flavus*. (**A**) *ZmAOS1*, (**B**) *ZmAOS2*, (**C**) *ZmOPR8*, and (**D**) *ZmACX*. Vertical bars indicate the standard error. Asterisk denotes significant upregulation/downregulation with 2-fold or higher FC using Welch’s *t*-test (* *p* ≤ 0.05; *** *p* ≤ 0.001).

The expression levels of the six maize *9-LOX* genes (*ZmLOX1*, *ZmLOX2*, *ZmLOX3*, *ZmLOX4*, *ZmLOX5,* and *ZmLOX12*), five maize *13-LOX* genes (*ZmLOX7*, *ZmLOX8*, *ZmLOX9*, *ZmLOX10*, and *ZmLOX11*), the lyase *ZmLOX6*, together with two oxylipin related encoding for allene oxide synthase 1 and 2 (*ZmAOS1* and *ZmAOS2*) and two genes involved in the synthesis of JA, 12-oxo-phytodienoic acid (12-OPDA) reductase (*ZmOPR8*), and acyl-CoA oxidase (*ZmACX*) were tested by reverse transcription-quantitative PCR (RT-qPCR) in Tzi18, W22, and Mo17 inbred lines and UFMu*lox4* mutant at 3 and 7 dpi with *F. verticillioides* and *A. flavus* (Figure 4, Figure 5, Figure 6, Figure 7, Figure 8, Figure 9 and Figure S3). Relative expression was calculated as the fold change (FC) of fungus- over mock-inoculated kernels. 

#### 2.3.1. Modulation of LOX and JA-Related Genes in Response to *Fusarium verticillioides*

As regards the *9-LOX* genes tested, *ZmLOX1*, *ZmLOX4,* and *ZmLOX5* showed significantly strong changes in gene expression for the inbred lines, while transcriptional changes were generally more attenuated or absent (FC = 1) in the mutant UFMu*lox4* (Figure 4A,D,E). The highest induction was observed for the genes *ZmLOX4* and *ZmLOX1* at 7 dpi in the genotype Mo17, with FC values of 37.9 and 25.2, respectively. Additionally, *ZmLOX4* showed the earliest upregulation at 3 dpi of about 7- and 4-fold for W22 and Tzi18, respectively, followed by a decrease thereafter. Similarly, its segmentally duplicated paralog, *ZmLOX5*, was significantly induced in Mo17 and W22 at 3 dpi. A general trend of downregulation or absence of differential expression was described for *ZmLOX2*, *ZmLOX3,* and *ZmLOX12* genes (Figure 4B,C,F). The gene *ZmLOX2* was significantly downregulated in Tzi18 at 3 dpi (FC = −2.3); the same line exhibited a significant transcript decrease for the gene *ZmLOX12* at 7 dpi along with the mutant UFMu*lox4* (FC of −2.6).

Concerning *13-LOX* and *ZmLOX6* genes, the highest induction was found for the inbred lines at 7 dpi (Figure 5A–C,F). *ZmLOX8* reached the strongest significant modulation in W22 (FC = 48.0), followed by Mo17 (FC = 3.7). In Tzi18, a weak induction was observed at 7 dpi but established earlier at 3 dpi (FC = 4.0). This trend was also observed for its segmentally duplicated paralog *ZmLOX7*, where an earlier transcriptional induction of 2.6-fold was found in this line. *ZmLOX7* was significantly overexpressed in Mo17 at 7 dpi too; this line showed the same pattern of expression for the gene *ZmLOX9*. Significant late upregulation was further achieved by the gene *ZmLOX6* in W22, showing an 18.1-fold induction. As for *9-LOX* genes, transcriptional changes were limited in UFMu*lox4*, which exhibited a significant downregulation for the gene *ZmLOX10* at 3 and 7 dpi (Figure 5D), whereas a slight upregulation occurred for *ZmLOX11* at 3 dpi (Figure 5E).

The expression of oxylipin- and JA-related genes was also affected by *F. verticillioides* inoculation (Figure 6). *ZmAOS1*, *ZmAOS2,* and *ZmACX1* were characterized by a significant earlier induction in Tzi18, where the induction of the latter gene was further extended at 7 dpi (Figure 6A,B,D). Transcript accumulation was also observed at 7 dpi in Mo17 for the genes *ZmAOS1* and *ZmACX1*, and in W22 for the gene *ZmAOS2*. Moreover, a peak of expression was reached at 3 dpi by the gene *ZmAOS1* in W22. Surprisingly, *ZmOPR8* showed limited or failing expression in all the genotypes at both dpi (Figure 6C).

#### 2.3.2. Modulation of LOX and JA-Related Genes in Response to *Aspergillus flavus*

The expression modulation of *ZmLOXs*, oxylipin-, and JA-related genes showed a lesser magnitude towards *A. flavus* inoculation compared to transcriptional changes induced by F*. verticillioides* (Figure 7, Figure 8, Figure 9 and Figure S3). In general, the most noteworthy differences were measured in the inbred lines, while no meaningful modulation was found in the UFMu*lox4* mutant. *A. flavus* treatment triggered a significant upregulation of maize *9-LOX* genes, *ZmLOX1,* and *ZmLOX4*, in a trend similar to that observed for *F. verticillioides* but to a lower extent (Figure 7A,D). In more detail, in inoculated Mo17 kernels, *ZmLOX1,* and *ZmLOX4* transcripts accumulated the greatest levels at 7 dpi, showing expression values of 11.2 and 9.4, respectively. Moreover, a significant induction of *ZmLOX4* was detected in Tzi18 and W22 at 3 dpi. Interestingly, *ZmLOX12* also exhibited enhanced FC of 6.2 in Mo17 at 7 dpi, whereas a significant downregulation of −4.0- and −3.5-fold was reported in Tzi18 and UFMu*lox4* mutant, respectively, as previously observed for *F. verticillioides* (Figure 7F). In addition, transcripts of *ZmLOX2* significantly decreased in Tzi18 at 7 dpi (Figure 7B). No significant differential expression was found for *ZmLOX3* and *ZmLOX5* genes (Figure 7C,E). 

As regards *13-LOX* genes, a significant *ZmLOX7* induction was observed in Tzi18 throughout the time course, while the upregulation was restricted to only 7 dpi in Mo17 (Figure 8A). A similar pattern was displayed by *ZmLOX8*, which showed a 2.5-fold induction for the line Tzi18 at 3 dpi and a 3.9-fold induction in Mo17 at 7 dpi (Figure 8B). The latter line presented the same trend of expression for the gene *ZmLOX9* (Figure 8C). Unlike *F. verticillioides* treatment, the level of *ZmLOX8* remained unchanged in W22 kernels inoculated with *A. flavus*. The expression of *ZmLOX10* and *ZmLOX11* was not significantly modulated for all genotypes and time points (Figure 8D,E). Moreover, the *ZmLOX6* gene expression pattern was similar between the two pathogens for the line W22 at 7 dpi (FC = 5.8), whereas a significant decrease in transcript accumulation was described for Tzi18 at the late time of inoculation (Figure 8F). 

Downstream enzymes in the 13-LOX pathway encoding for *ZmAOS1* and *ZmACX* were also differentially regulated upon *A. flavus* inoculation (Figure 9). *ZmAOS1* was significantly upregulated in Tzi18 early at 3 dpi (FC = 2.1), whereas its induction was delayed to 7 dpi in Mo17 (Figure 9A). As for *F. verticillioides*, *ZmACX* transcripts consistently accumulated in Tzi18 throughout the time course, while its overexpression was limited to 7 dpi in Mo17 inoculated kernels (Figure 9C). In contrast, both *ZmAOS2* and *ZmOPR8* remained unaffected by pathogens in all conditions assayed (Figure 9B,D).

### 2.4. Analysis of Maize Lipid Peroxidation

**Figure 10:**
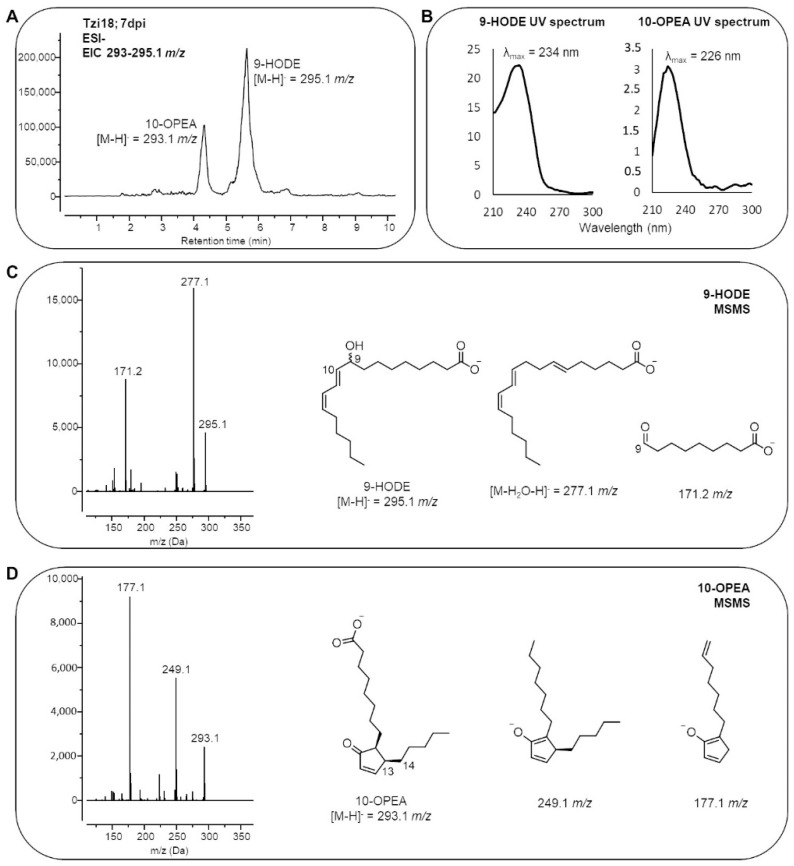
MS/MS and UV characterisation of oxylipins. (**A**) Extracted ion chromatogram of 293–295.1 *m*/*z* of Tzi18 at 7 dpi. (**B**) UV spectra of 9-HODE and 10-OPEA. (**C**,**D**) MS/MS fragmentation of 9-HODE and 10-OPEA. The structure of diagnostic daughter ions are reported.

**Figure 11:**
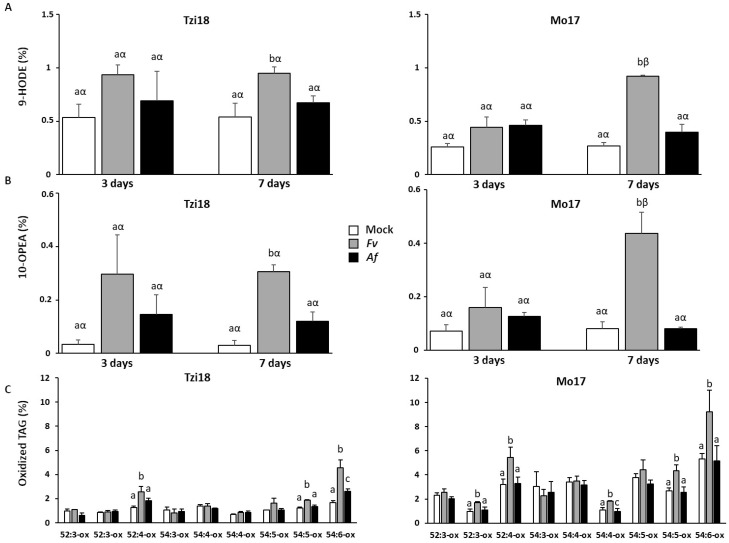
Free fatty acid and triacylglycerol derived oxylipin profiling of the two maize genotypes Tzi18 and Mo17 upon *F. verticillioides* and *A. flavus* inoculation. (**A**,**B**) Kinetics of 9-HODE and 10-OPEA accumulation in mock and inoculated kernels at 3 and 7 dpi in Tzi18 and Mo17 genotypes. Vertical bars indicate the standard error and the different letters over the histograms indicate significant differences among the means of the three treatments (mock, Fv, and Af) within each treatment time (3 and 7 days; Latin letters) and between the two times of inoculation, 3 and 7 dpi for each fungus (Greek letters), as resulting from Tukey multiple comparison and Student’s *t*-test (*p* ≤ 0.05), respectively. (**C**) Relative distribution of oxidized TAGs in mock and inoculated kernels at 7 dpi in Tzi18 and Mo17 genotypes. The vertical bars indicate the standard error and the different letters over the histograms indicate significant differences among the means of the three treatments (mock and 7 dpi with Fv and Af) as resulting from the Tukey multiple comparison test (*p* ≤ 0.05).

To gain further insight into the activity of ZmLOXs during plant interaction with *F. verticillioides* and *A. flavus*, total lipids were extracted from mock and inoculated kernels at 3 and 7 dpi and analyzed to detect and describe peroxidation events and their oxylipin profiles. Preliminary analysis did not show any relevant accumulation of free fatty acids between mock and treated samples in W22 and UFMulox4 mutants. Conversely, a more pronounced modulation was observed in Tzi18 and Mo17. As a result, additional research was conducted in these two genotypes (Figure 10 and Figure 11). 

Concerning the free fatty acids fraction, linoleic (18:2) acid was the most abundant species in the kernels with an average relative quantity of 52.4% and 51.3% in Tzi18 and Mo17, respectively, while linolenic (18:3) acid was quantified in lower amounts (3.0% and 1.8%, respectively) (Table S2). Focusing on the oxidized species accumulating upon fungal inoculation, two major species were detected: the 9-hydroxy-10,12-octadecadienoic acid (9-HODE) and 10-OPEA (Figure 10A). The former is derived from the reduction of 9-hydroperoxy-10,12-octadecadienoic acid by triphenylphosphine, added during the extraction to reduce to alcohol the hydroperoxyl form. The regiospecificity of the oxidative event was assessed by MS/MS spectrometry: fragmentation occurring at the C9-C10 bond generated the characteristic daughter ion of 171.1 *m*/*z*, which unambiguously established the regiochemical position of the –OH function at C9 of linoleic acid (Figure 10C). The identity of 10-OPEA, which has a mass of 295.1 *m*/*z* and is identical to the hydroxy-10,12,15-octadecatrienoic acid (hydroxy-18:3), was suggested by its UV absorbance at 226 nm (Figure 10B), which differs from the conjugated diene absorbance peak at 234 nm. Due to its characteristic fragmentation pattern (Figure 10D), MS/MS analysis confirmed the 293.1 *m*/*z* peak to be 10-OPEA. In more detail, it was observed that the decarboxylation and subsequent cleavage of the C13-C14 bond with the formation of the daughter ions 249.1 and 177.1 *m*/*z*, respectively. 9-HODE and 10-OPEA both derive from the 18:2 peroxidation catalyzed by one or more maize 9-LOXs. Upon *F. verticillioides* inoculation, both compounds accumulated in Tzi18 and Mo17 but with different kinetics. Conversely, although the two species were measured after *A. flavus* treatment, their accumulation was not significant (Figure 11A,B). In Tzi18, the level of 9-HODE and 10-OPEA rapidly increased by about 2- and 10-fold, respectively, at 3 dpi and then remained constant over the 7 dpi with *F. verticillioides*. Instead, Mo17 instead showed a delayed response, between 3 and 7 dpi, reaching similar peroxidation levels. 

Concerning membrane and esterified lipids, galactolipids, and triacylglycerols (TAG) were investigated at 7 dpi. While galactolipids did not show significant changes in peroxidation upon fungal infections (Table S3), TAGs were more interesting due to an increase in the oxidized forms of those species containing polyunsaturated chains, represented exclusively by 18:2 chains. The TAGs’ relative abundance showed that 52:3, 52:4, 54:4, 54:5, and 54:6 were the most represented species in the analyzed maize kernels (Table S4). The oxidized TAG profiles of the studied genotypes at 7 dpi are illustrated in Figure 11C. Tzi18 showed a generally lower basal level of TAG peroxidation compared to Mo17, but upon fungal inoculation, Tzi18 significantly accumulated 52:4 and 54:6 oxidized forms (2- and 2.7-folds, respectively) in response to *F. verticillioides* and to a lower extent (1.5-fold) in response to *A. flavus*. Interestingly, these species have two and three 18:2 chains, but only single-chain oxidized forms were detected. Differently, Mo17 accumulated 52:4 and 54:6 oxidized forms only in response to *F. verticillioides*, the final proportion being higher than Tzi18 due to the more elevated basal oxidation level.

## 3. Discussion

This study aimed to provide knowledge of the ZmLOX allozyme role in maize artificially inoculated with the mycotoxigenic fungi *F. verticillioides* and *A. flavus*. 

The availability of a growing number of maize genome sequences organized in a repository like maizeGDB [40], which provides suitable tools for genomic analysis, allowed us to perform an in silico survey of the intraspecific genetic variability of the ZmLOX family in 31 genomes. The pan-genome analyses highlighted that a substantial portion of the variation underlying adaptive traits is genotype-specific, thus making it mandatory to evaluate genomic diversity when studying the genetic architecture of complex traits [44,45].

At the protein level, the analysis of the alignments computed for each of the 13 allozymes underlined a high heterogeneity and many interesting features. First, in a few cases, some of the 9-LOX isoforms (ZmLOX2, 3, 4, and 12) were absent in a restricted group of lines; this phenomenon is referred to as PAV and represents an extreme form of copy number variation, which contributes to the high genetic and phenotypic diversity of maize [44,46,47]. An opposite case of copy number variation is represented by ZmLOX3 duplication in PH207. More frequently, the occurrence of 9- and 13-putatively non-functional proteins were observed, due to the loss of the final isoleucine residue, which is essential for iron coordination in the active site [48]. Notably, ZmLOX6 and ZmLOX13 lacked the N-terminal PLAT/LH2 domain in all the genotypes. Such a large structural difference was also found for ZmLOX5, 12, and 7, but only in ten genotypes. This plasticity is likely made possible by the high redundancy observed in the gene family in maize, especially in the case of 9-LOXs, where mutant lines in one or two *ZmLOX* genes did not suppress completely the phenotype [29], except for *ZmLOX3* [33]. Second, not all the allozymes showed the same degree of sequence variability. In fact, ZmLOX1, 2, 4, 8, 9, and 10 showed a high similarity index, whereas extensive sequence divergences were observed for ZmLOX3, 5, 7, and 13, especially for the M37W and PH207 lines. This outcome was in line with a previous study where large gene-order and gene structural variations were observed in PH207 compared to B73 and Mo17 genomes [49]. Many *ZmLOX* genes are likely derived from tandem or segmental duplication followed by transposable element insertion and possible excision [36]. Examples are the following groups originating from duplication events: ZmLOX1-2, ZmLOX3-4-5, ZmLOX7-8, and ZmLOX10-11. It is supposed that during evolution, natural selection pressure maintained at least one functional gene per group, allowing mutation accumulation on the others to increase genetic variability. The high conservation level observed for ZmLOX8 and 10 correlated very well with previous functional studies showing that they are necessary, and not replaceable by others, for JA and GLV synthesis [26,50,51].

Finally, the tissue specificity gene expression of the different *ZmLOX* isoforms has been estimated through the mining of the transcriptomic data available for 22 of the NAM lines. Interestingly, the three considered organs, root, shoot, and seed, showed quite different *ZmLOX* profiles. The root exhibited the overall highest *ZmLOX* modulation, in particular the 9-LOXs: oxylipins produced through these pathways could contribute to plant interaction and defense with soil microbial communities, as reported for other phytoalexins, such as stilbenoids [52], terpenes [53], and camalexins [54]. In the shoot, *ZmLOXs* involved in the synthesis of GLVs, such as *ZmLOX10* and *6*, were more transcribed compared to other organs. In the seed, *ZmLOX* modulation was lower, except for the three 9-LOX, *ZmLOX1*, *2,* and *3*. A more elevated constitutive level of these isoforms could be related to a higher resistance to specific pathosystems as well as to the fast induction of other isoforms, such as *ZmLOX4*, *5*, and *12* [36].

This preliminary in silico analysis not only highlighted the high level of diversity of ZmLOXs among different genotypes but also conserved features both in the protein sequence and gene expression profiles, which can support the interpretation of the experimental results collected during the inoculation experiments presented here. 

The two pathosystems were first discussed separately, because it was observed that the same genotypes responded differently to the two pathogens. This behavior was previously demonstrated by Gao et al. in the *lox3* mutant inoculated with *F. verticillioides* and *Aspergillus* spp. [33,34], and by Lanubile et al. [55], who observed an increased H_2_O_2_ content only in kernels of resistant and susceptible genotypes after *A. flavus* infection. The production of H_2_O_2_ could cause tissue necrosis and enhance the necrotrophic lifestyle of this pathogen. On the other hand, inoculations of *F. proliferatum* and *F. subglutinans* did not affect the accumulation of this compound [55]. 

As regards the pathosystem *F. verticillioides*-maize, Tzi18 showed a reduced growth of the pathogen with limited spread and low fumonisin contamination at 7 dpi, confirming the resistance patterns previously observed towards Fusarium seedling rot [38]. An early upregulation of the genes *ZmLOX4*, *7,* and *8* and a downregulation of *ZmLOX2* and *6*, along with an early induction of *ZmAOS1*, *2*, and *ZmACX*, was also detected. Concerning oxylipin accumulation, Tzi18 accumulated 9-HODE and 10-OPEA already at 3 dpi and exhibited TAG peroxidation at 7 dpi. From this data, it seems more likely that resistance was related to 10-OPEA synthesis via *ZmLOX4* and *ZmAOS* activities. However, JA synthesis with the involvement of *ZmLOX8* and *ZmACX* genes cannot be excluded. *ZmLOX6* induction was reported to occur during compatible interactions of maize after treatment with a virulent strain of the fungal pathogen *Cochliobolus carbonum* just before massive tissue collapse [28]. In contrast, infection with the avirulent strain resulted in notably lower transcript accumulation [28]. This finding suggests that *ZmLOX6* expression is more likely involved in disease development than in the defense response.

Concerning Mo17, intermediate levels of FER symptoms and fumonisin accumulation were measured. Early transcript induction was found only for *ZmLOX5*, whereas other genes such as *ZmLOX1*, *4*, *7*, *8*, *9*, as well as *ZmAOS1* and *ZmACX* were modulated later at 7 dpi, coherently with the kinetics of 9-HODE and 10-OPEA accumulation as well as TAG peroxidation. This delayed reaction of genes and oxidized lipids suggests a similar response as described for Tzi18, probably mediated by 10-OPEA and possibly JA.

On the other hand, W22 presented extensive fungal growth to neighboring kernels and an elevated content of fumonisins. Early induction of genes *ZmLOX4*, *5*, *8*, and *AOS1* at 3 dpi and late upregulation of *ZmLOX6* and *8* at 7 dpi were observed. 

In a more remarkable way, the mutant UFMu*lox4* was the most susceptible to FER and accumulated the highest concentrations of fumonisins. A very contained differential expression of *ZmLOX* genes was revealed, consisting of the slight upregulation of *ZmLOX11* at 3 dpi and the downregulation of *ZmLOX10* at 3 and 7 dpi along with *ZmLOX12* at 7 dpi. These results agreed with those reported by Lanubile et al. [38] where, despite different physiological stages (seedling vs. ear) and UFMu*lox4* mutants (10924 vs. 01831) being considered, the loss of *ZmLOX4* was demonstrated to severely impair the resistance to *F. verticillioides*. Likewise, Battilani et al. [39] reported increased susceptibility of *ZmLOX4* knock-out mutants as compared to the wildtype B73, providing evidence that this isoform is essential in the response to *F. verticillioides* infection. 

Upon *A. flavus* inoculation, Tzi18 was less affected by the pathogen compared to the other inbred lines and showed a lower aflatoxin content. Therefore it can be considered moderately resistant to this pathogen, as previously observed [56]. At 7 dpi, there was an early upregulation of genes ZmLOX4, 7, and 8, as well as ZmAOS1 and ZmACX, and a downregulation of ZmLOX6 and 12. In addition, 9-HODE and 10-OPEA were already accumulated at 3 dpi and maintained at 7 dpi, despite in a not significant way compared to mock kernels and to a lower extent than treatment with *F. verticillioides*. Moreover, the accumulation of oxidized TAGs was not observed with the exception of the compound 54:6. Once again, *ZmLOX4* and *ZmAOS1* were promptly induced and could be responsible for the low amount of 10-OPEA, which could have an important inhibitory effect against *A. flavus,* which was shown to be more sensitive to this phytoalexin compared to *F. verticillioides* [29]. Additionally, the downregulation of *ZmLOX6* suggests that the plant was still actively counteracting the fungal attack.

An enhanced production of aflatoxins was recorded in Mo17 that exhibited only late transcriptional changes of the genes *ZmLOX1*, *4*, *12*, *7*, *8*, *9*, *ZmAOS1,* and *ZmACX*, most of them to a lower magnitude than against *F. verticillioides*. No synthesis of 9-HODE and 10-OPEA was detected as well as oxidized TAGs. A slight attempt to produce these two phytoalexins was made by the line, but the fungus prevailed. It can be suggested that the more delayed and attenuated gene expression due to a late recognition of the pathogen failed in oxylipin production, making Mo17 more susceptible to *A. flavus* as compared to *F. verticillioides*. This hypothesis will need to be better supported by additional investigations into the apparently contrasting roles of these two pathogens.

W22 and its mutant, which showed an extensive fungal growth and aflatoxin accumulation, did not display any significant modulation except for *ZmLOX4* and *6* upregulation in W22. 

It is interesting to compare results from this work with those reported for the same pathosystem, involving resistant (Mp719) and susceptible (Va35) genotypes [36]. Analogously to Tzi18, an upregulation of *ZmLOX4*, *7,* and *8* was observed at 3 dpi in field conditions, together with *ZmLOX13*, which is not evident in this study. Additionally, Mo17 showed the same modulation but at 7 dpi, supporting the hypothesis that resistance mechanisms could be related to the fast kinetics of pathogen recognition and response. Further evidence in support of the kinetic importance was provided by a recent inoculation study of the susceptible B73 genotype and its *lox5-3* mutant line, and of a resistant genotype W438 with *F. graminearum*: the very complete oxylipin profiling showed again that 9-LOX derived oxylipins, among which 10-OPEA, were already accumulated at 12 h post-inoculation (hpi) in the resistant line, compared to the others [32]. Conversely, 13-LOX derived oxylipins, among which JA and its derived forms, were anti-correlated with resistance. In the same study, all the 9-LOX isoforms were significantly modulated either at 12 or 24 hpi, making it difficult to identify direct correlations between single genes and metabolites. 

In summary, the employment of the mutant UFMu*lox4* supports the conclusion that *ZmLOX4* plays a strategic role in controlling defense responses and in developing kernels, as its loss is detrimental [38,39]. Due to 9-LOX redundancy, the early induction of *ZmAOS1* in the resistant genotype Tzi18 appears to be a more promising marker of breeding for resistance. *ZmAOS1* and *ZmAOS2* represent candidate genes for the initial steps in pathogen-induced death acid biosynthesis, as 10-OPEA and a series of related 14- and 12-carbon metabolites are responsible for cytotoxicity and consequent cell death [29]. Further biochemical and genetic experiments will be needed to confirm this hypothesis. 

Conversely, *ZmLOX6* upregulation could be considered a marker of susceptibility, which can also be exploited via a gene editing approach. In agreement with this, Gao et al. [28] reported that the induction of *ZmLOX6* was more likely responsible for disease development rather than for its containment functioning as a susceptibility factor to several fungal pathogens. Further studies on *lox6* edited mutants generated in our laboratory are already ongoing to characterize their function.

## 4. Materials and Methods

### 4.1. In Silico Analysis of the Maize Pan-Genome and Pan-Transcriptome of LOX Isoforms

The PLAT/LH2 domain (PF01477) and lipoxygenase (PF00305) Hidden Markov Model (HMM) profiles were obtained using the Pfam database (http://pfam.xfam.org, accessed on 31 January 2022) and probabilistic HMM profiles were used to query lipoxygenase protein sequences against the representative maize genome Zm-B73-REFERENCE-NAM-5.0 (RefGen_v5; https://www.ncbi.nlm.nih.gov/genome/annotation_euk/Zea_mays/103/, accessed on 31 January 2022) by using the phmmer command line of HMMER version 3.3.2. The respective homologous protein sequences of all ZmLOX allozyme groups of the pan-genome were retrieved from maizeGDB (https://www.maizegdb.org/gene_center/gene, accessed on 31 January 2022). Multiple protein sequence alignments of each LOX allozyme of the pan-genome were generated using MUSCLE (https://www.ebi.ac.uk/Tools/msa/muscle/, accessed on 31 March 2022) and further used for Blosum62 global similarity and phylogenetic analysis. Protein sequence similarity was calculated using SIAS (http://imed.med.ucm.es/Tools/sias.html, accessed on 31 March 2022) and the average overall similarity index score of genotypes was used to plot the distribution of sequence similarity in the pan-genome.

FASTA format protein sequences of each ZmLOX allozyme were subjected to Batch CD-Search in the Conserved Domain Database (CDD) (https://www.ncbi.nlm.nih.gov/Structure/bwrpsb/bwrpsb.cgi, accessed on 30 April 2022) against Pfam v33.1-18271 PSSMs with default search parameters and E-value cutoff at 0.01. The retrieved domain annotations were visualized using TBtools software’s graphic domain pattern illustrator (https://github.com/CJ-Chen/TBtools/releases, accessed on 30 April 2022). 

Basal transcriptomic abundances of root, shoot, and whole seeds at 20 days after pollination were compared using the maize RNA-seq expression platform, qTeller (https://qteller.maizegdb.org/genes_by_name_NAM.php, accessed on 30 April 2022). Transcript per million (TPM) data for respective ZmLOX genes were downloaded, and mean values with 95% confidence intervals (CI) were plotted to visualize basal expression plasticity across B73 and NAM founder lines.

### 4.2. Description of the Maize Inbred Lines and UFMulox4 Mutant

**Figure 12:**
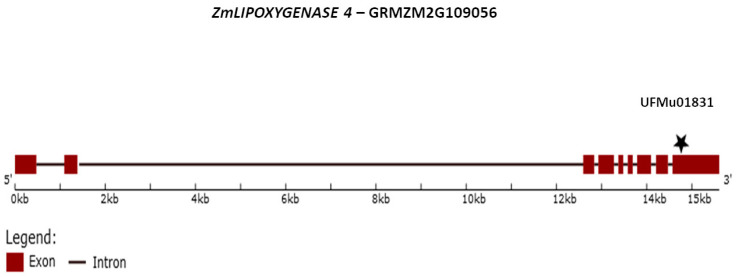
Mutator element insertion site for UFMu01831 in the *ZmLIPOXYGENASE 4* gene model of inbred line W22.

Three maize inbred lines Tzi18, Mo17, and W22 belonging to the “Goodman” maize association panel were used in this study [16,17,57]. Seeds were obtained from the USDA-ARS-NCRPIS (Iowa State University, Regional Plant Introduction Station, Ames, Iowa, USA, 50011-1170). Tzi18 was previously reported as resistant to Fusarium stalk and seedling rot [16,38,57], while Mo17 was described as moderately resistant to FER [58]; W22 was found susceptible to Fusarium seedling rot [38]. Moreover, Tzi18 and Mo17 were described to be mildly resistant [56] and susceptible [59] to aflatoxin accumulation, whereas concerning W22, levels of resistance/susceptibility against *A. flavus* have not been reported yet. The UFMu*lox4* mutant (UFMu01831) belongs to the UniformMu population, and seeds were obtained from the Maize Cooperation Stock Center using online tools maintained at MaizeGDB.org. Mutator-transposable element insertional mutagenesis of the UFMu*lox4* mutant was in the genetic background of the line W22. The mutant was introgressed into in W22 background with three backcrosses, and homozygous F_3_ mutant plants were used (BC_3_F_3_) [38]. The mutant UFMu01831 had a Mu insertion in exon 9 (Figure 12), as previously reported, and was susceptible to Fusarium seedling and kernel rot [38,39,60]. Seeds of inbred lines and mutants were maintained by sibbing at the Department of Sustainable Crop Production, Università Cattolica del Sacro Cuore, Piacenza, Italy.

### 4.3. Set-Up of Inoculation Assay

The inoculation assay was carried out at the experimental site of the Department of Sustainable Crop Production, Università Cattolica del Sacro Cuore, Piacenza, Italy. The sowing was made on 16 April 2021. Experimental units were single-row plots of 5 m in length, separated by 0.80 m. Three rows were sown for each maize genotype. Plots were overplanted and thinned at the three- to four-leaf stage to 20 plants. Standard agronomic practices for growing maize were followed. Fertilizer rates were applied as follows: 250 kg/ha N, 100 kg/ha P_2_O_5_, and 80 kg/ha K_2_O. Irrigation was applied by a drip system in order to prevent water stress. Maize ears were hand-pollinated starting on 16 June.

The inoculum was prepared from *F. verticillioides* (ITEM 10027) and *A. flavus* (ITEM 8069) strains, supplied by the Institute of Sciences of Food Production, National Research Council, Bari, Italy. Conidial suspensions were set up as described in [61], obtaining a final concentration of 10^6^ conidia/mL and stored at 4 °C before use. 

Maize ears were inoculated at kernel milk stage (R3), 15 days after hand-pollination, according to the pin-bar inoculation method [11,62]. The primary ear of 6–9 plants per plot was inoculated. *F. verticillioides* and *A. flavus* were inoculated in separate plots. Control ears were inoculated with sterilized deionized water (mock-inoculated). The inoculated and immediately adjacent kernels taken from the same ear were collected at 3- and 7-days post-inoculation (dpi). Three biological replicates were prepared for each time point, where each replicate was derived from the pool of kernels of two to three plants. Before further use, the collected kernels were ground in liquid nitrogen with a pestle and mortar and stored at −80 °C. 

FER and AER severity were visually evaluated, assessing the percentage of the rotted surface of the ear, using a 7-point severity grid and assigning 1 for the absence of infection, and numbers from 2 to 7 according to the percentage level of the infection, where 2 = 1–3%, 3 = 4–10%, 4 = 11–25%, 5 = 26–50%, 6 = 51–75%, and 7 = 76–100% [14].

### 4.4. Analysis of Mycotoxin Content

Total fumonisins (B_1_ + B_2_ + B_3_) and aflatoxins (B_1_ + B_2_ + G_1_ + G_2_) were determined in kernels collected at 7 dpi with *F. verticillioides* and *A. flavus* using VICAM Fumo-V and Afla-V AQUA strips, respectively (VICAM, Watertown, MA, USA). One g of cryo-grinded kernel samples was transferred into a 50 mL tube and 25 mL of AQUA extraction buffer (VICAM) was added. The mixture was vigorously vortexed for 2 min at maximum speed, followed by a centrifugation step at 8000× *g* for 1 min. Samples were further filtered through 100 mm Whatman filter paper and collected into plastic cups. The quantification of fumonisins and aflatoxins was carried out by transferring 100 μL of the extracted lysate into Fumo-V and Afla-V AQUA strips (VICAM) by dropping at ~1 drop/second. The strips were kept for 5 min on a flat surface and were inserted into the Vertu reader (VICAM), where the results were read. Values of fumonisins and aflatoxins given in the text were expressed in ppm and ppb, respectively.

### 4.5. RNA Extraction and Real-Time RT-qPCR Gene Expression Analysis

Total RNA isolation and purification were performed according to [63]. DNA contamination was removed with Amplification Grade DNase I according to the supplier’s instructions (Sigma-Aldrich, St. Louis, MO, USA). The extracted RNA was quantified using a fluorometric assay (Qubit, Invitrogen, Carlsbad, CA, USA) and the integrity was checked using gel electrophoresis. 

Complementary DNA (cDNA) was synthesized from 2 µg of total RNA using a High-Capacity cDNA Reverse Transcription Kit (Thermo Fisher Scientific, Inc., Waltham, MA, USA). Reverse transcription-quantitative PCR (RT-qPCR) experiments were performed using the FluoCycle™ II SYBR Green master mix (EuroClone S.p.a., Milan, Italy) and the CFX-96 device (Bio-Rad, Hercules, CA, USA). For real-time RT-qPCR, 20 ng of single-strand cDNA was determined using a fluorometric assay (Qubit, Thermo Fisher Scientific). Relative RT-qPCR was performed under the following conditions: 95 °C for 3 min and 40 cycles at 95 °C for 15 s, specific annealing temperatures for 30 s, followed by a melting curve analysis. The NCBI primer design tool (https://www.ncbi.nlm.nih.gov/tools/primer-blast/, accessed on 31 January 2022) was used to create gene-specific ZmLOX primers, and their specificity was confirmed using melting curve analysis (Table S5). Zea mays *β-actin* and *ubiquitin-conjugating enzyme* (UBCP) reference genes were used to normalize the expression level of the target genes [38,64]. The efficiency of primers was determined using linreg [65] and Cycle thresholds (Ct) were obtained for three biological replicates with duplicate technical replicates. Fold changes (FC) values of gene expression were calculated using the 2^−ΔΔCt^ method [66] and calibrated on the mock-inoculated kernels. FC values were also used to construct an expression heatmap using the pheatmap R package [67].

### 4.6. Lipid Analysis

Total lipids were extracted using a modified Bligh and Dyer method as described by [68]. Extraction was carried out from 500 mg of freeze-grounded kernels collected from maize ears at 3 and 7 dpi with *F. verticillioides* and *A. flavus* and their respective mock samples, considering three biological replicates. Extracted aliquots were dried under nitrogen flux, weighed, and stored at −20 °C.

The lipid extract was analyzed by liquid chromatography-mass spectrometry (LC-MS) (Model 1100 series; Hewlett-Packard; Palo Alto, CA, USA) coupled to a quadrupole ion-trap mass spectrometer (Esquire LCTM; Bruker, Bremen, Germany) equipped with an electrospray ionization source in negative ion modes. Chromatographic separation of lipids was carried out at 303 K using a C18 column (TMC18; length, 100 mm; particle size, 2.6 μm; internal diameter, 2.1 mm; pore size, 100 Å; Phenomenex, Torrence, CA, USA). The solvent system consisted of eluant A as MeOH/H_2_O (7:3, *v*/*v*) containing 10 mM ammonium acetate and eluant B as isopropanol/MeOH (10:90, *v*/*v*) containing 10 mM ammonium acetate. Samples were resuspended in MeOH:CHCl_3_ (4:1, *v*/*v*) to a final concentration of 1 mg/mL, and 10 µL were run with a linear gradient from 65% eluant B to 100% in 40 min, plus 20 min of isocratic 100% B at 1 mL/min to elute the diglycerides and triglycerides. The column was then equilibrated to 65% eluant B for 10 min. The MS scan range was 13,000 U/s in the range from 50 to 1500 *m*/*z*, with a mass accuracy of ~100 ppm. The nebulizer gas was high purity nitrogen at a pressure range of 20–30 psi, at a flow rate of 6 L/min, and at 300 °C. The electrospray ionization was operated in negative ion mode for the analysis of free fatty acids. A positive mode was operated for the analysis of galactolipids and triglycerides (TAG). For the class assignment and relative intra-class molar distribution of lipid species, the extracted ion chromatograms of the positive and/or negative ion full scan data were integrated using the Data Analysis 3.0 software (Bruker Daltonik, Bremen, Germany). The relative percentage of peroxidation of 18:2, galactolipids, and TAGs was calculated as the ratio of the absolute ESI area (negative or positive, respectively) of the extracted ion current (EIC) of each oxidized product with respect to the ESI (+) area of the EIC of total (native and oxidized) 18:2 or corresponding galactolipid and TAG species. The regiochemistry of the 18:2 peroxidation and the identification of 10-OPEA were established using either short-wavelength UV-DAD or MS/MS detection.

### 4.7. Statistical Analysis

Mycotoxin content, gene expression, and lipid analysis were carried out on three biological replicates and plotted values represent the respective means and standard error of the mean (SEM). Statistical analysis of mycotoxin accumulation and lipid metabolites was carried out on log-normalized ln [y + 1] mycotoxin concentration and lipid relative abundance data, respectively, using one-way analysis of variance (ANOVA) followed by Tukey’s multiple comparison test and Student’s *t*-test at *p* ≤ 0.05. Gene expression log_2_ NRQ values of mock and inoculated kernels were discriminated against using Welch’s unequal variance *t*-test (*p* ≤ 0.05). The statistical package IBM SPSS statistics 27 (IBM Corp., Armonk, NY, USA) was used for data analysis. 

## Data Availability

Data is contained within the article or Supplementary Material.

## Supplementary Materials

The supporting information can be downloaded at: https://www.mdpi.com/article/10.3390/ijms231810894/s1.
