# Supplementary material for: Functional Study of Lipoxygenase-Mediated Resistance against Fusarium verticillioides and Aspergillus flavus Infection in Maize
Source: Int J Mol Sci. 2022 Sep 17;23(18):10894. doi: 10.3390/ijms231810894 (PMC9503958; doi:10.3390/ijms231810894)
Supplement: Supplementary file 1 [file ijms-23-10894-s001.zip › ijms-1878051-supplementary.pdf]

Figure S1. Overview of protein domain architecture of 9-LOX allozymes in the maize pan-genome using MUSCLE generated alignments and CDD. The protein sequences were retrieved from the B73_v5 reference genome, the 25 NAM founder genomes, and Mo17, W22, PH207, EP1 and F7 line genomes. Yellow and green rectangles represent PLAT/LH2 (PF01477) and lipoxygenase domain (PF00305), respectively. Stars (*) indicate the absence of the C-terminal isoleucine. (**A**) ZmLOX1: highly conserved in all the genotypes considered, except PH207, which is truncated at the C-terminal, thus lacking the Fe-coordinating isoleucine with a likely loss of functionality. (**B**) ZmLOX2: highly conserved in all the genotypes considered, but absent in PH207. (**C**) ZmLOX3: a tandem duplication occurs in PH207; premature C-terminal truncation (no isoleucine, likely non-functional) in Ky21 and Ki3; absent in Tx303. (**D**) ZmLOX4: absent in PH207, CML277 and CML333 genotypes. (**E**) ZmLOX5: B97, CML247, P39 and Tzi8 genotypes lack the PLAT/LH2 domain, while PH207 is prematurely truncated at the C-terminal (no isoleucine, likely non-functional). (**F**) ZmLOX12: absent in CML228 and CML247 genotypes; incomplete and non-functional in W22; premature C-terminal truncation in Mo17.

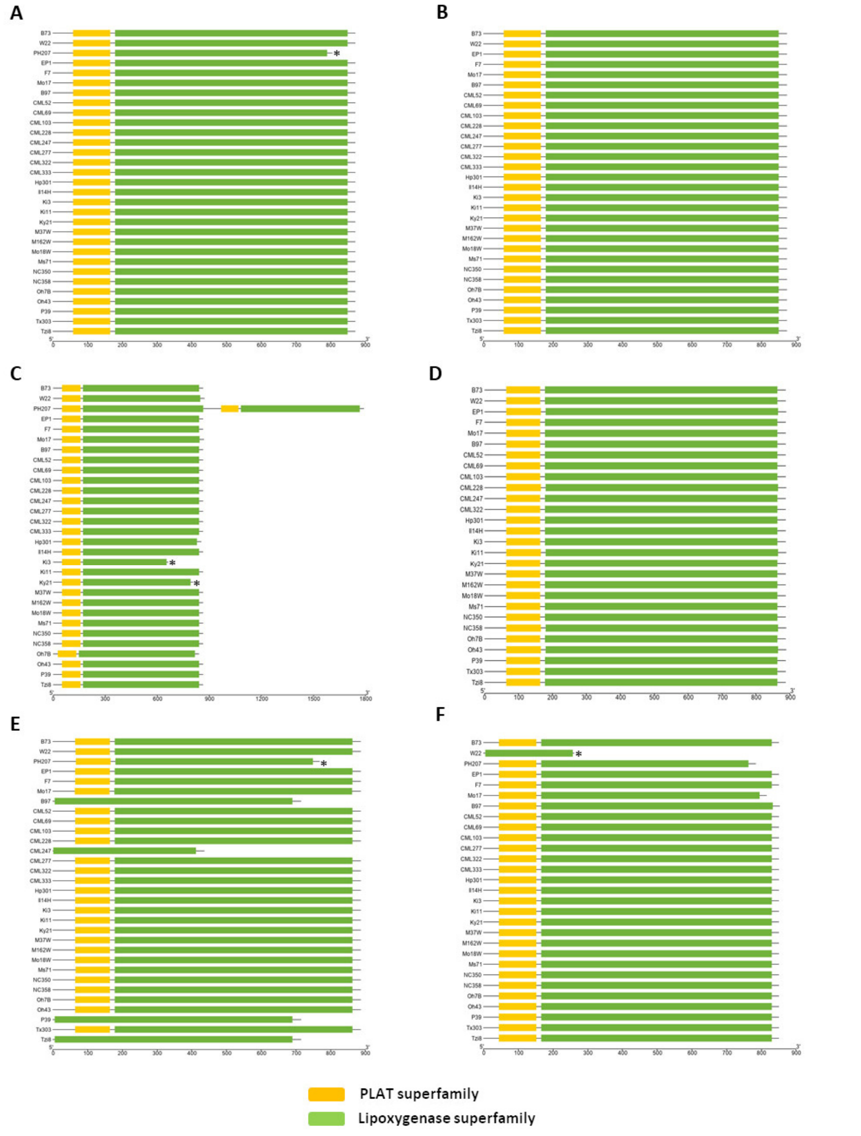

Figure S2. Overview of protein domain architecture of 13-LOX allozymes and ZmLOX6 lyase in the maize pan-genome using MUSCLE generated alignments and CDD. The protein sequences were retrieved from the B73_v5 reference genome, the 25 NAM founder genomes, and Mo17, W22, PH207, EP1 and F7 line genomes as above. Yellow and green rectangles represent PLAT/LH2 (PF01477) and lipoxygenase domain (PF00305), respectively. Stars (*) indicate the absence of the terminal isoleucine. (**A**) ZmLOX6: all genotypes lack PLAT/LH2 domain; a C-terminal fusion with other domains, such as FGART-AT_N (PF18076), FGART-AT_linker (PF18072), PurM-like (PF02769), and GAT_1 (PF13507), indicated with different colors in the figure, is present in Mo17. (**B**) ZmLOX7: CML333, PH207, Il14H and M37W genotypes lack PLAT/LH2 domain. The C-terminal isoleucine is missing in B97, M37W and P39 (likely non-functional). (**C**) ZmLOX8: PH207 lacks PLAT/LH2 domain and is prematurely truncated at the C-terminal (no isoleucine, likely non-functional). (**D**) ZmLOX9: highly conserved domain structure, the C-terminal isoleucine is missing in PH207 (likely non-functional). (**E**) ZmLOX10: highly conserved domain structure in all genotypes. (**F**) ZmLOX11: highly conserved domain structure, prematurely truncated at the C-terminal (no isoleucine, likely non-functional) in Mo71. (**G**) ZmLOX13: PLAT/LH2 domain is absent in all genotypes.

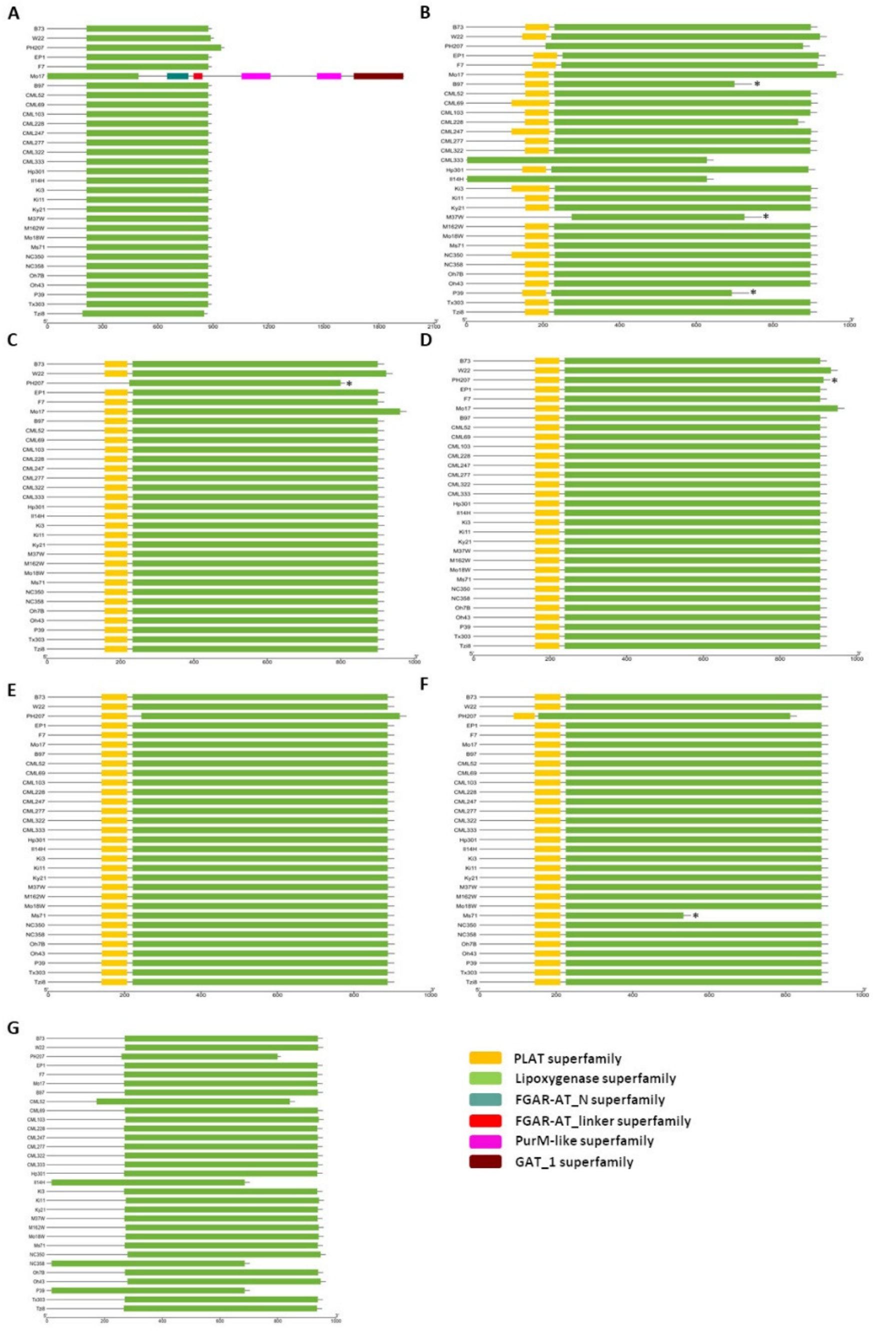

Figure S3. Heat map and clustering of differentially expressed *ZmLOX*, oxylipin- and JA-related genes grouped in two clusters based on fungal inoculation (*F. verticillioides* and *A. flavus*) at 3 and 7 dpi in Tzi18, Mo17, W22 and UFMu*lox4* lines. The color scale indicates fold change (FC) values (red and blue refer to upregulated and downregulated genes, respectively). The heat map and clustering of FC expression values were generated with pheatmap R package.

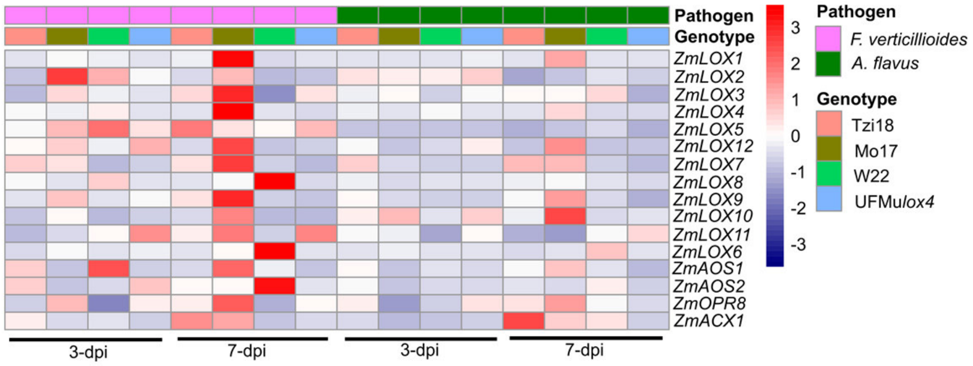

Table S1. Percentage (%) of Fusarium and Aspergillus ear rot (FER and AER, respectively) severity for Tzi18, Mo17, W22 and UFMu*lox4* at 7 dpi.

	**Tzi18**	**Mo17**	**W22**	**UFMu*lox4***
**FER %**	3.50 ± 0.5	25.50 ± 2.3	50.60 ± 4.5	65.00 ± 5.4
**AER %**	5.70 ± 1.4	55.00 ± 3.5	51.50 ± 3.5	71.40 ± 5.7

Table S2. Distribution (%) of the total fatty acid fraction in Tzi18 and Mo17 at 7 dpi.

Maize Inbred line Tzi18

Fatty acid fraction	Chromatogram	Distribution of the total fatty acid fraction (%)						Genotype average (%)	
		mock	SD	*A. flavus*	SD	*F. verticillioides*	SD	average	SD
14:0	227.3	0,28	0,21	0,03	0,00	0,05	0,03	0,12	0,14
18:3	277.3	3,71	0,51	2,62	0,11	2,68	0,81	3,00	0,61
16:1	253.3	0,14	0,03	0,10	0,01	0,14	0,09	0,13	0,02
18:2	279.3	54,06	2,63	52,35	0,89	50,78	3,78	52,40	1,64
16:0	255.3	27,10	0,44	19,39	0,35	22,06	2,60	22,85	3,91
18:1	281.3	9,70	0,97	22,52	1,36	20,82	6,67	17,68	6,97
18:0	283.3	4,71	1,68	2,56	0,05	2,83	0,56	3,37	1,17

Maize Inbred line Mo17

Fatty acid fraction	Chromatogram	Distribution of the total fatty acid fraction (%)						Genotype average (%)	
		mock	SD	*A. flavus*	SD	*F. verticillioides*	SD	average	SD
14:0	227.3	0,14	0,07	0,03	0,01	0,04	0,01	0,07	0,06
18:3	277.3	2,36	0,21	1,40	0,14	1,61	0,14	1,79	0,51
16:1	253.3	0,25	0,11	0,13	0,01	0,16	0,01	0,18	0,06
18:2	279.3	51,53	2,78	51,91	2,93	50,31	3,77	51,25	0,84
16:0	255.3	23,49	1,44	16,34	0,96	17,25	1,44	19,03	3,89
18:1	281.3	16,74	0,83	26,24	1,91	25,94	1,69	22,97	5,40
18:0	283.3	5,31	1,70	3,69	0,61	4,00	0,80	4,33	0,86

Table S3. Distribution (%) of galactolipids in Tzi18 and Mo17 at 7 dpi.

Maize Inbred line Tzi18

Galactolipids	Extracted Ion (m/z)	Distribution of galactolipids (%)						Genotype average (%)	
		mock	SD	*A. flavus*	SD	*F. verticillioides*	SD	average	SD
Mono 36:6	797.7	6,12	0,62	6,94	0,60	7,64	0,50	6,90	0,76
Mono 36:5	799.7	7,98	0,82	6,54	0,49	7,24	0,13	7,25	0,72
Mono 36:4	801.7	18,53	0,40	18,88	0,88	17,49	0,44	18,30	0,73
Mono 34:3	775.7	0,81	0,05	0,63	0,19	2,86	3,59	1,43	1,24
Mono 34:2	777.7	2,03	0,73	2,20	0,35	2,15	0,43	2,13	0,09
Di 36:6	959.7	3,58	0,99	3,47	0,06	3,55	0,37	3,53	0,06
Di 36:5	961.7	8,92	0,54	8,60	0,64	8,49	0,37	8,67	0,22
Di 36:4	963.7	22,84	1,50	23,67	1,57	22,86	0,90	23,12	0,47
Di 36:3	965.7	6,65	0,24	6,12	0,64	4,16	3,12	5,64	1,31
Di 36:2	967.7	1,69	0,32	2,14	0,12	2,17	0,43	2,00	0,27
Di 36:1	969.7	0,26	0,04	0,30	0,10	0,31	0,15	0,29	0,03
Di 34:3	937.7	3,27	0,34	3,31	0,21	3,23	0,38	3,27	0,04
Di 34:2	939.7	14,43	0,35	14,20	0,64	14,75	0,80	14,46	0,28
Di 34:1	941.7	2,42	0,04	2,65	0,43	2,72	0,31	2,60	0,15

Maize Inbred line Mo17

Galactolipids	Extracted Ion (m/z)	Distribution of galactolipids (%)						Genotype average (%)	
		mock	SD	*A. flavus*	SD	*F. verticillioides*	SD	average	SD
Mono 34:2	777.7	1,68	0,76	1,26	0,36	1,14	0,52	1,36	0,28
Mono 34:3	775.7	0,61	0,11	0,49	0,05	0,57	0,01	0,56	0,06
Mono 36:4	801.7	17,95	0,53	17,71	0,38	17,47	0,26	17,71	0,24
Mono 36:5	799.7	6,42	0,07	5,90	0,31	6,37	0,07	6,23	0,29
Mono 36:6	797.7	4,92	0,20	5,64	0,21	7,49	0,77	6,02	1,33
Di 34:1	941.7	2,95	0,08	3,12	0,27	3,12	0,23	3,07	0,10
Di 34:2	939.7	14,82	0,40	14,91	0,20	13,86	0,65	14,53	0,58
Di 34:3	937.7	2,75	0,24	2,69	0,15	2,66	0,17	2,70	0,05
Di 36:1	969.7	0,26	0,09	0,37	0,10	0,37	0,16	0,34	0,07
Di 36:2	967.7	2,21	0,50	2,27	0,30	2,21	0,42	2,23	0,03
Di 36:3	965.7	10,12	0,72	9,63	0,42	8,61	0,42	9,46	0,77
Di 36:4	963.7	23,70	0,22	24,54	0,87	23,88	0,70	24,04	0,44
Di 36:5	961.7	8,35	0,53	8,07	0,31	7,91	0,40	8,11	0,22
Di 36:6	959.7	2,94	0,08	3,12	0,41	4,10	0,30	3,38	0,62

Table S4. Distribution (%) of TAG species in Tzi18 and Mo17 at 7 dpi.

Maize Inbred line Tzi18

TAG species	Extracted Ion (m/z)	Distribution of TAG species (%)						Genotype average (%)	
		mock	SD	*A. flavus*	SD	*F. verticillioides*	SD	average	SD
50:1	855.7; 850.7	2,76	0,08	2,86	0,19	3,04	0,35	2,89	0,14
50:2	853.7; 848.7	4,01	0,14	4,12	0,06	4,06	0,06	4,06	0,05
50:3	851.7; 846.7	0,70	0,02	0,75	0,01	0,80	0,04	0,75	0,05
50:4	849.7; 844.7	0,22	0,01	0,28	0,00	0,26	0,02	0,26	0,03
52:1	883.8; 878.8	0,70	0,06	1,32	0,13	1,39	0,50	1,13	0,38
52:2	881.8; 876.8	7,83	0,24	7,83	0,76	7,88	1,10	7,85	0,03
52:3	879.8; 874.8	10,86	0,42	10,52	0,30	10,42	0,14	10,60	0,23
52:4	877.8; 872.8	11,38	0,36	11,29	0,53	10,78	1,69	11,15	0,32
52:5	875.8; 870.8	2,74	0,23	3,20	0,09	3,08	0,14	3,01	0,24
52:6	873.8; 868.8	0,28	0,05	0,41	0,05	0,42	0,03	0,37	0,08
54:1	911.8; 906.8	0,43	0,08	0,77	0,04	0,73	0,24	0,64	0,18
54:2	909.8; 904.8	1,84	0,22	2,23	0,14	2,49	0,56	2,18	0,33
54:3	907.8; 902.8	9,02	0,22	8,65	0,78	8,94	1,41	8,87	0,19
54:4	905.8; 900.8	12,53	0,31	12,08	0,72	11,98	0,58	12,20	0,29
54:5	903.8; 898.8	13,35	0,58	12,48	0,61	12,06	0,97	12,63	0,66
54:6	901.8; 896.8	11,42	0,41	11,37	0,96	10,86	1,66	11,22	0,31
54:7	899.7; 894.8	3,27	0,27	4,00	0,43	3,74	0,36	3,67	0,37
56:2	937.8; 932.8	0,84	0,14	0,74	0,06	0,88	0,15	0,82	0,07
56:3	935.8; 930.8	1,33	0,14	1,09	0,07	1,30	0,10	1,24	0,13
56:4	933.8; 928.8	1,52	0,16	1,25	0,02	1,48	0,10	1,42	0,15
56:5	931.8; 926.8	0,84	0,15	0,66	0,03	0,73	0,11	0,74	0,09
58:3	963.8; 958.8	0,41	0,08	0,35	0,01	0,43	0,05	0,40	0,04
58:4	961.8; 956.8	0,40	0,12	0,35	0,02	0,41	0,02	0,39	0,03
58:5	959.8; 954.8	0,08	0,02	0,07	0,01	0,07	0,02	0,07	0,01

Maize Inbred line Mo17

TAG species	Extracted Ion (m/z)	Distribution of TAG species (%)						Genotype average (%)	
		mock	SD	*A. flavus*	SD	*F. verticillioides*	SD	average	SD
50:1	855.7; 850.7	1,27	0,02	1,54	0,08	1,58	0,10	1,46	0,17
50:2	853.7; 848.7	3,11	0,23	3,31	0,11	3,24	0,03	3,22	0,10
50:3	851.7; 846.7	0,57	0,08	0,67	0,09	0,71	0,11	0,65	0,08
50:4	849.7; 844.7	0,37	0,02	0,39	0,06	0,40	0,07	0,38	0,01
52:1	883.8; 878.8	0,75	0,07	1,16	0,26	1,09	0,15	1,00	0,22
52:2	881.8; 876.8	5,50	0,21	5,89	0,22	5,80	0,17	5,73	0,20
52:3	879.8; 874.8	9,60	0,09	9,66	0,06	9,36	0,03	9,54	0,16
52:4	877.8; 872.8	12,54	0,52	11,45	0,74	11,66	1,17	11,88	0,57
52:5	875.8; 870.8	2,53	0,08	2,61	0,18	2,69	0,16	2,61	0,08
52:6	873.8; 868.8	0,27	0,01	0,29	0,03	0,34	0,08	0,30	0,04
54:1	911.8; 906.8	0,38	0,05	0,67	0,16	0,56	0,11	0,54	0,15
54:2	909.8; 904.8	2,17	0,26	2,54	0,39	2,51	0,29	2,41	0,21
54:3	907.8; 902.8	7,82	0,39	7,86	0,45	7,75	0,50	7,81	0,05
54:4	905.8; 900.8	12,62	0,19	12,21	0,27	11,83	0,15	12,22	0,39

54:5	903.8; 898.8	13,81	0,58	13,72	0,78	12,75	1,01	13,43	0,59
54:6	901.8; 896.8	14,04	0,56	13,46	1,39	13,65	1,30	13,72	0,30
54:7	899.7; 894.8	2,79	0,04	2,85	0,14	2,94	0,14	2,86	0,08
56:2	937.8; 932.8	0,80	0,12	0,89	0,13	0,91	0,17	0,87	0,06
56:3	935.8; 930.8	1,33	0,16	1,40	0,21	1,36	0,24	1,36	0,04
56:4	933.8; 928.8	2,04	0,16	2,06	0,26	2,01	0,29	2,04	0,02
56:5	931.8; 926.8	1,39	0,09	1,27	0,09	1,22	0,19	1,29	0,09
58:3	963.8; 958.8	0,36	0,05	0,43	0,08	0,42	0,11	0,40	0,03
58:4	961.8; 956.8	0,54	0,06	0,59	0,13	0,62	0,14	0,58	0,04
58:5	959.8; 954.8	0,07	0,01	0,08	0,01	0,08	0,02	0,08	0,01

Table S5. Primer sequences for real-time RT-qPCR analyses.

Gene	Primer forward	Primer reverse	Gramene ID B73_v5/B73_v3	Source
ZmLOX1	CTGCTCTACCCCAACACCTC	GAACGGGGAAACGCAAACAA	Zm00001eb144930<br>GRMZM2G156861	NM_001111533.2<br>This paper
ZmLOX2	AGCAGAGCTAGCGGTGATTC	CAGCACCAGTACACCAAGGA	Zm00001eb144960<br>GRMZM2G156861	NM_001112503.2<br>This paper
ZmLOX3	TCAAGATCGAGGGCAAGGTG	CTGTGGTCAGAGGTGTTGGG	Zm00001eb054040<br>GRMZM2G109130	NM_001112045.1<br>This paper
ZmLOX4	CACCGGACACTCCCTGAGA	ACGTCCAGCACCTCCTTCTT	Zm00001eb054050<br>GRMZM2G109056	NM_001112504.2<br>This paper
ZmLOX5	GGAACTTCACGGACCTTGCT	TCCTTTATCGCCAGCTCCAC	Zm00001eb216870<br>GRMZM2G102760	NM_001112505.1<br>This paper
ZmLOX6	GGAACTTCACGGACCTTGCT	TCCTTTATCGCCAGCTCCAC	Zm00001eb067710<br>GRMZM2G040095	NM_001112506.1<br>This paper
ZmLOX7	GCGAATGCGAGCTGTTTTGA	CGAACGATCTCCCCAGTAGC	Zm00001eb423430<br>GRMZM2G070092	NM_001361937.1<br>This paper
ZmLOX8	CCAGCTGTTCCAGAAGATCCC	ATGGATCATGGACGGAGGAG<br>G	Zm00001eb081610<br>GRMZM2G104843	DQ335766.1<br>This paper
ZmLOX9	ACTGGCGGTTCGACCTAGAG	CTGCACATACGACTCCACCC	Zm00001eb005920<br>GRMZM2G017616	NM_001112507.1<br>This paper
ZmLOX10	CATCGACGAGTGCAACAACA	TGGACTAATGCATGCTGAGGA	Zm00001eb206040<br>GRMZM2G015419	NM_001112510.2<br>This paper
ZmLOX11	GTACGAACTGCTCAAGCCCT	TGCACGCAATAAGGCAAACC	Zm00001eb236430<br>GRMZM2G009479	NM_001112511.1<br>This paper
ZmLOX12	TCCATTACGGAAGCGCACAT	CGCAGGTATGGCATCAGGTA	Zm00001eb134020<br>GRMZM2G106748	NM_001112527.1<br>This paper
ZmAOS1	ACCGGTGTCACGAAAGCTAC	CCGAGGGTGAGAGAGAAATC	Zm00001d048021<br>GRMZM2G067225	NM_001111774.2<br>This paper
ZmAOS2	TGATCGACCGTCCGTCAAAG	AGCAGGGGCAAACCAAACA	Zm00001eb009340<br>GRMZM2G002178	NM_001152442.2<br>This paper
ZmOPR8	ATCTCCGAGGGCACCATCAT	TCCCTTGGCATGAACAGCAT	Zm00001eb177330<br>GRMZM2G082087	NM_001112363.2<br>This paper
ZmACX	TGGAAGGACCCTCTGAACGA	AGGTCGCGTGAGATTTTCTGA	Zm00001eb376020<br>GRMZM5G864319	NM_001136902.1<br>This paper
ZmUBCP	GACCGACAGGGCCAAGTATG	CCGGCACATTCACCAGTTTG	Zm00001eb066940<br>GRMZM2G102471	NM_001154750.1<br>This paper
Zmβ-actin	ATGGTCAAGGCCGGTTTCG	TCAGGATGCCTCTCTTGGCC	Zm00001d012277<br>GRMZM2G320797	AY273142.1<br>Maschietto et al. 2015
